# Anti-parasitic drug discovery against *Babesia microti* by natural compounds: an extensive computational drug design approach

**DOI:** 10.3389/fcimb.2023.1222913

**Published:** 2023-08-16

**Authors:** Shopnil Akash, Md. Eram Hosen, Sajjat Mahmood, Sumaiya Jahan Supti, Ajoy Kumer, Shamima Sultana, Sultana Jannat, Imren Bayıl, Hiba-Allah Nafidi, Yousef A. Bin Jardan, Amare Bitew Mekonnen, Mohammed Bourhia

**Affiliations:** ^1^ Department of Pharmacy, Faculty of Allied Health Sciences, Daffodil International, University, Dhaka, Bangladesh; ^2^ Professor Joarder DNA and Chromosome Research Laboratory, Department of Genetic Engineering and Biotechnology, University of Rajshahi, Rajshahi, Bangladesh; ^3^ Department of Microbiology, Jagannath University, Dhaka, Bangladesh; ^4^ Department of Genetic Engineering and Biotechnology, University of Rajshahi, Rajshahi, Bangladesh; ^5^ Laboratory of Computational Research for Drug Design and Material Science, Department of Chemistry, European University of Bangladesh, Dhaka, Bangladesh; ^6^ Department of Pharmaceutical Sciences, School of Health and Life Sciences. North South University, Dhaka, Bangladesh; ^7^ Department of Pharmacy, International Islamic University Chittagong, Chittagong, Bangladesh; ^8^ Department of Bioinformatics and Computational Biology, Gaziantep University, Gaziantep, Türkiye; ^9^ Department of Food Science, Faculty of Agricultural and Food Sciences, Laval University, Quebec, QC, Canada; ^10^ Department of Pharmaceutics, College of Pharmacy, King Saud University, Riyadh, Saudi Arabia; ^11^ Department of Biology, Bahir Dar University, Bahir Dar, Ethiopia; ^12^ Department of Chemistry and Biochemistry, Faculty of Medicine and Pharmacy, Ibn Zohr University, Laayoune, Morocco

**Keywords:** molecular docking, DFT, molecular dynamic simulation, ADMET, *B. microti*, and drug design

## Abstract

Tick-borne Babesiosis is a parasitic infection caused by *Babesia microti* that can infect both animals and humans and may spread by tick, blood transfusions, and organ transplantation. The current therapeutic options for *B. microti* are limited, and drug resistance is a concern. This study proposes using computational drug design approaches to find and design an effective drug against *B. microti*. The study investigated the potentiality of nine natural compounds against the pathogenic human *B. microti* parasite and identified Vasicinone and Evodiamine as the most promising drugs. The ligand structures were optimized using density functional theory, molecular docking, molecular dynamics simulations, quantum mechanics such as HOMO–LUMO, drug-likeness and theoretical absorption, distribution, metabolism, excretion, and toxicity (ADMET), and pharmacokinetics characteristics performed. The results showed that Vasicinone (−8.6 kcal/mol and −7.8 kcal/mol) and Evodiamine (−8.7 kcal/mol and −8.5 kcal/mol) had the highest binding energy and anti-parasitic activity against *B. microti* lactate dehydrogenase and *B. microti* lactate dehydrogenase apo form. The strongest binding energy was reported by Vasicinone and Evodiamine; the compounds were evaluated through molecular dynamics simulation at 100 ns, and their stability when they form complexes with the targeted receptors was determined. Finally, the pkCSM web server is employed to predict the ADMET qualities of specific molecules, which can help prevent negative effects that arise from taking the treatment. The SwissADME web server is used to assess the Lipinski rule of five and drug-likeness properties including topological polar surface area and bioavailability. The Lipinski rule is used to estimate significant drug-likeness. The theoretical pharmacokinetics analysis and drug-likeness of the selected compounds are confirmed to be accepted by the Lipinski rule and have better ADMET features. Thus, to confirm their experimental value, these mentioned molecules should be suggested to carry out in wet lab, pre-clinical, and clinical levels.

## 1 Introduction

Babesia are intra-erythrocytic protozoa that belong to the same species of Apicomplexa as Plasmodia. Hard-bodied ticks spread them worldwide and can infect several vertebrates, including humans (Bloch et al., 2019). Among the more than 100 Babesia species, *B. microti* is predominant can infect humans, and is prevalent throughout the world. It is a significant cause of endemic illness in China and the United States (US) (Kumar et al., 2021). *B. microti* is primarily transmitted by *Ixodes scapularis*, also known as the deer tick or black-legged tick (Curcio et al., 2016). Additionally, they can spread through blood transfusions and, less commonly, through organ transplants and prenatal transfer (Puri et al., 2021).

The most acute disease caused by the parasite is babesiosis, in which RBC is infected and destroyed. Fever (up to 41°C or 106°F), arthralgia, anorexia, nausea, headaches, and exhaustion are the most frequent symptoms. According to the severity, physical examination results may include hepatosplenomegaly, ocular hemorrhage, and pharyngeal erythema. Hemolytic anemia and thrombocytopenia may be consistent with typical laboratory results (Vanniere, 2008; Ortiz et al., 2020; Bastos et al., 2021). Babesia parasites in red blood cells in a blood smear can be detected under a microscope to make the diagnosis (Ortiz et al., 2020; Singh et al., 2023).

In 1888, a Hungarian pathologist and microbiologist named Victor Babes discovered that intraerythrocytic bacteria were the root of febrile hemoglobinuria in cattle (Babes, 1888; Vannier and Krause, 2012). Approximately 50 years later, the first known human case of babesiosis was discovered, and a splenectomized Croatian herdsman who had died of infection later was associated with *B. divergens* (Skrabalo and Deanovic, 1957; Vannier and Krause, 2012). In 1969, on Nantucket Island, off the coast of Massachusetts, the first case of *B. microti* infection was discovered in an immunocompetent person (Western et al., 1970; Vannier and Krause, 2012).

There has been an increase in *B. microti* infections worldwide in recent decades. More than 2,000 instances are reported yearly in the US, although the actual number is likely far higher. Human babesiosis has the most significant incidence in areas with high temperatures. The most dominating species*, B. microti*, is indigenous to southwestern China, and the northeastern and northern mid-western US (Vannier and Krause, 2012; Zhou et al., 2014; Fang et al., 2015; Krause, 2019; Kumar et al., 2021). In many parts of the world, babesiosis is now acknowledged as an emerging health concern. To date, babesiosis is categorized as a nationally notifiable disease and is acknowledged as a rising health issue in many parts of the world (Jones et al., 2008; Aguero-Rosenfeld, 2011; Vannier and Krause, 2012).

Human infections with *B. microti* may manifest clinically, ranging from asymptomatic infection to a severe, rapid deadly disease (Gorenflot et al., 1998). About one in five cases of babesiosis spread by blood transfusions has fatal consequences. Immunocompetent people can be infected with *B. microti* for up to 2 years without developing symptoms. *B. microti* parasitemia has been found in immunocompetent hosts for over a year who received a standard course of antibiotic therapy for more than 2 years in untreated patients (Krause et al., 1998; Moritz et al., 2016; Abraham et al., 2018). Immunocompromised patients, such as those with asplenia, HIV/AIDS, cancer, or who are on immunosuppressive medications, frequently have severe diseases that can return up to 2 years even after the treatment (Bloch et al., 2019). The severity of the illness is much higher and linked to an increased death rate in those without a spleen, as it is the first immunological barrier for babesia after transmission to the human body (Rosner et al., 1984; Krause et al., 1998; Zintl et al., 2003; Mebius and Kraal, 2005; Krause et al., 2007; Vannier and Krause, 2012; Bloch et al., 2019).

The treatment options for disease caused by *B. microti* include clindamycin and quinine or atovaquone and azithromycin (the preferred drug combination) (Kumar et al., 2021). However, it can produce resistance to even these two most standard combinations of drugs. The resistance to atovaquone and azithromycin is due to genetic mutation of the targeting protein of the drugs. Atovaquone’s target is the Babesia mitochondrial electron transport chain’s cytochrome B protein, while azithromycin’s target is the apicoplast protein, which prevents protein translation in these organelles (Lemieux et al., 2016; Simon et al., 2017; Bloch et al., 2019).

Due to the drug resistance and lack of a strong and permanent cure and to avoid the side effects of using broad-spectrum high-power antibiotics, the search for new drugs with minimal toxicity and high effectiveness is still ongoing. The introduction of novel therapeutic interventions, such as new anti-parasitic medications or drug combinations, enhanced anti-parasitic drug duration strategies, or immunoglobulin preparations, and also novel preventative techniques, can reduce the health burden of chronic and recrudescent babesiosis (Abraham et al., 2018; Bloch et al., 2019).

However, searching for a new drug requires a lot of resources, time, and cost to perform pre-clinical or clinical trials. Still, this does not ensure that the drugs will be available to the market. Since many adverse/side effects and toxicity are revealed during clinical or pre-clinical trials, advanced computational drug design approaches are necessary to find and design an effective drug against *B. microti.* This method is the best choice for fast hit identification and hit-to-lead selection against a specific pathogen or disease. Also, the time efficiency and low cost are the advantages of this effective approach to finding and designing a new pharmacologically active substance. Moreover, before going to an experimental study, computational investigation may determine the theoretical toxicity, which can also reduce the chance of drug failure during the experimental studies (Kapetanovic, 2008).

Alkaloids are a class of nitrogenous organic compounds that have a numerous range of physiological activity. The primary function of alkaloids in plants is toxin production against diseases and predators. Although plant alkaloid toxicity can be diverse, it frequently involves neurotoxicity or disruption of the cell signaling cascade. The same toxic characteristics of the plant’s defense mechanism are frequently exploited while searching for new drugs. Particular toxicities of some alkaloids may be utilized to fight certain cancerous cell types or to eradicate certain pests or microorganisms [25]. For this study, we have selected a few natural alkaloids as potential inhibitors of *B. microti* for the rapid development of effective and sustainable therapeutic options targeting the pathogen. In addition to numerous other activities expressed by alkaloids, they demonstrated anti-inflammatory, anticancer, analgesic, local anesthetic and pain alleviation, neuropharmacological, antibacterial, and antifungal effects, and they are also included as vitamins and diet ingredients (Kurek, 2019).

## 2 Materials and methods

### 2.1 Molecular optimization by density functional theory

**Figure 1:**
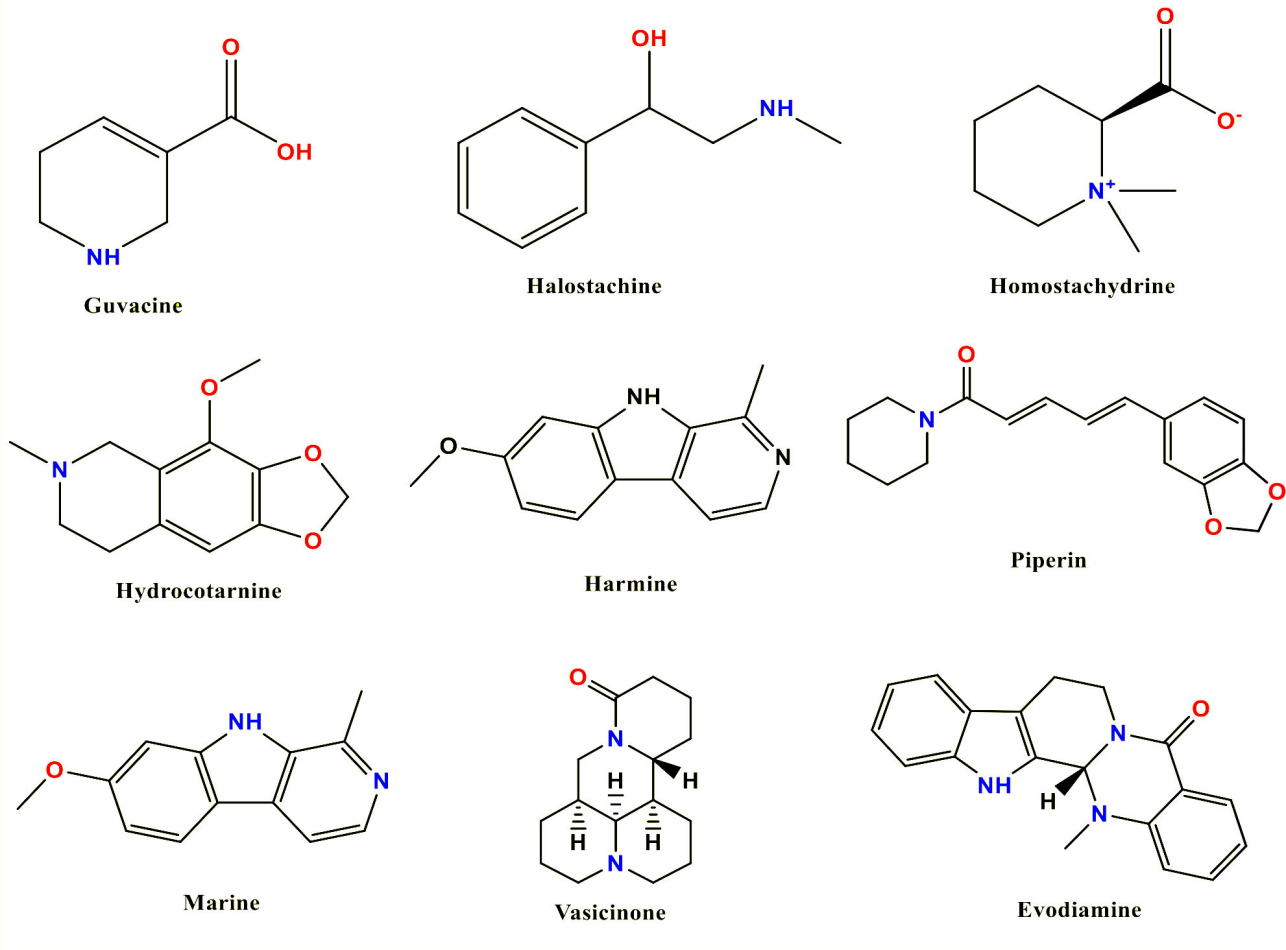
Molecular structures of selected compounds.

Selected alkaloid’s SDF-formatted structure files were fetched from the PubChem database for optimization (Kim et al., 2019). To achieve the configuration with the lowest ground-state energy, we optimized the geometrical arrangement of the molecules and rearranged the atomic components using density functional theory (DFT) (Orio et al., 2009). This is essential since molecular docking and molecular dynamics (MD) simulation demand ligands with an optimal geometrical arrangement. For geometry optimization, all ligand structures were imported to the Gaussian 09W program and the optimization procedure was completed at DFT/B3LYP/6-31G (d’p’) levels (Rajalakshmi et al., 2015; Cao et al., 2018). After optimization, the electron acceptor and electron donor properties were determined by calculating the frontier orbital energies, including the highest occupied molecular orbital (HOMO) and the lowest unoccupied molecular orbital (LUMO), as well as the energy gap, hardness, and softness. These characteristics also provide information regarding the chemical reactivity and stability of compounds (Kumer et al., 2019). Finally, optimization molecules were saved in pdb format and again uploaded in the Material Studio program to design three-dimensional structures. **Figure 1** displays the 2D chemical structure (Sharma et al., 2019).

### 2.2 Protein preparation and molecular docking study and visualization

**Figure 2:**
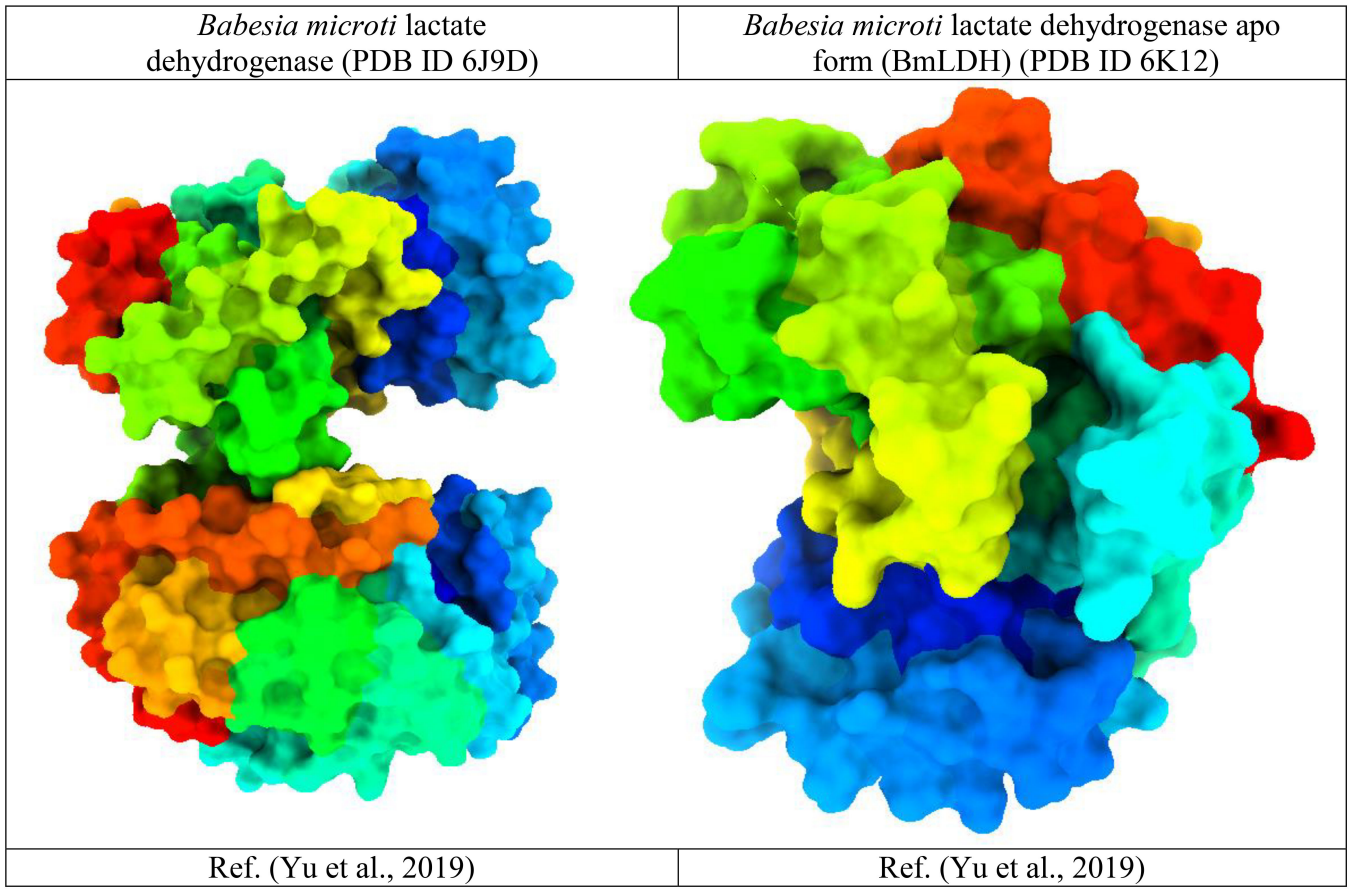
Three-dimensional protein structure of the *Babesia microti*.

To investigate and evaluate the molecular interactions between proteins and ligands at their binding sites, several bioinformatics approaches have been established. In our study, we used molecular docking, a bioinformatics tool that is extensively employed in medicinal chemistry research for the development of new therapeutic molecules that target key virulence factors for particular diseases (Vilar et al., 2008). Polar hydrogens and Kohlman charges were added before docking, and they were followed by the removal of water molecules and previously attached undesirable compounds to make it more straightforward for the ligand to be docked at its most advantageous location (**Figure 2**). We implemented the docking technique using PyRx free package v0.8 integrated with AutoDock Vina, assuming the entire protein surface is a receptor (Dallakyan et al., 2015). The program’s exhaustiveness parameter was set at 8 to obtain the most optimal docking position with the lowest possible margin of error. Ligands were perceived as flexible during docking. However, proteins have been perceived as stiff. Ultimately, protein-ligand docked compounds have been investigated and visualized using Pymol software and the Discovery studio program.

### 2.3 Determination of ADMET, Lipinski rule, and pharmacokinetics

Predicting ADMET qualities for certain lead compounds during the development of innovative drugs helps to prevent any negative effects that arise from taking the treatment (Kesharwani et al., 2020). By excluding compounds with subpar ADMET characteristics from the pipeline for medication development, this strategy can also reduce overall costs. As a result, we have estimated the critical ADMET features for our selected compounds using the pkCSM web server (https://biosig.lab.uq.edu.au/pkcsm/prediction), which works with novel graph-based signatures by employing a predictive model to compute the ADMET values for specific molecules (Pires et al., 2015). This platform is recognized for providing an efficient assessment of pharmacokinetic and toxicological characteristics to reflect the chemistry and structure of medicinal compounds. Target molecules have been loaded into the pkCSM query bar using the standard Simplified Molecular Input Line Entry System (SMILES), and the anticipated critical ADMET characteristics were recorded.

On the other hand, the commonly applied Lipinski’s rule of five is a fantastic guideline for assessing the pharmacological and biological activity of drug-like compounds (Lipinski, 2004). As a result, we assessed the Lipinski rule through the SwissADME web server (http://www.swissadme.ch/) along with estimating significant drug-like characteristics like Topological Polar Surface Area (TPSA) and bioavailability (Daina et al., 2017).

### 2.4 Molecular dynamics simulation

Yet Another Scientific Artificial Reality Application (YASARA) dynamics software was used to perform MD simulation using Assisted Model Building with Energy Refinement (AMBER) 14 force field (Wang and Wolf, 2004; Krieger and Vriend, 2014). The docked complexes were the earliest cleanup and optimized, and optimization of the hydrogen bond network was also performed. GAFF (general AMBER force field) and assigning AM1BCC charges were used to generate the topology files of ligands. A cubic simulation cell was created by the TIP3P solvation model having a periodic boundary condition (Harrach and Drossel, 2014). To neutralize the simulated system, the physiological conditions were set at 0.9% NaCl, 310 K, and pH 7.4 (Krieger and Vriend, 2015). To determine the long-range electrostatic interaction with an 8.0-Å cutoff radius, the particle mesh Ewald (PME) method was used (Essman et al., 1995). A time step of 2.0 fs was chosen for the simulation. By using simulated annealing techniques, the primary energy minimization was carried out using the steepest gradient algorithms (5,000 cycles). From the simulated trajectories, the RMSD, Rg, SASA, hydrogen bond, and Molecular Mechanics/Position-Boltzmann Surface Area (MMPBSA) binding free energy were evaluated. The simulation was conducted for 100 ns, and the simulation trajectories were captured after every 100 ps (Cheung and Sundram, 2017; Baildya et al., 2021; Patil et al., 2021).

## 3 Results and discussion

### 3.1 Lipinski rule and pharmacokinetics

The structure files of selected ligands and standard Diminazene were uploaded to the SwissADME web server to generate their respective canonical SMILES so that these SMILES can be used to obtain respective pharmacokinetics characteristics and Lipinski’s rule statement.

According to the results generated by SwissADME, all of the selected nine alkaloids and Diminazene follow Lipinski’s rule with no violations (**Table 1**). The rule states that the molecular weight for any drug-like molecules must not cross 500 Daltons. We can observe that all of our selected alkaloids have molecular weights of <500 Daltons. Moreover, none of the compounds has more than five hydrogen bond donors; in fact, only Diminazene presented five hydrogen bond donors, whereas the remaining compounds have a maximum of 2 hydrogen bond donors (Guvacine and Halostachine). In addition, the number of hydrogen bond acceptors for all the compounds did not cross 10, which is acceptable according to the Lipinski rule. Ultimately, Consensus Log Po/w values for potential lead molecules were also assessed and were found to be in an acceptable range. The TPSA is an effective method to estimate polar surface area since it eliminates the requirement to determine the necessary biological conformation or conformations or determine the drug’s three-dimensional arrangement (Prasanna and Doerksen, 2009). However, a TPSA score less than 140 Å² is ideal for drug-like molecules; here, all our selected alkaloids followed this threshold (Prasanna and Doerksen, 2009). Finally, the bioavailability scores were also calculated, revealing that the selected nine alkaloid compounds and Diminazene had acceptable (0.55) bioavailability scores.

**Table 1:** Data of Lipinski rule.

Name	Molecular weight	Hydrogen bond acceptor	Hydrogen bond donor	Topological polar surface area (Å²)	Consensus Log *p*_o/w_	Lipinski rule		Bioavailability Score
						Result	violation	
Guvacine	127.14	3	2	49.33	−0.3	Yes	0	0.55
Halostachine	151.21	2	2	32.26	0.97	Yes	0	0.55
Homostachydrine	157.21	2	0	40.13	−1.4	Yes	0	0.55
Hydrocotarnine	221.25	4	0	30.93	1.73	Yes	0	0.55
Harmine	212.25	2	1	37.91	2.78	Yes	0	0.55
Piperin	285.34	3	0	38.77	3.03	Yes	0	0.55
Matrine	248.36	2	0	23.55	1.8	Yes	0	0.55
Vasicinone	202.21	3	1	55.12	0.94	Yes	0	0.55
Evodiamine	303.36	1	1	39.34	2.7	Yes	0	0.55
Standard Diminazene	281.32	4	5	136.49	1.86	Yes	0	0.55

### 3.2 Molecular docking analysis against targeted receptor of *Babesia microti*

Pharmacological research and development must comprehend the mechanisms by which small-molecule ligands identify and engage with proteins. Therefore, molecular docking is most frequently used to achieve this objective as it offers investigations of important molecular processes, such as ligand binding mechanisms and the related intermolecular interactions that maintain the ligand–receptor complex (Ferreira et al., 2015). *Babesia microti* lactate dehydrogenase and *B. microti* lactate dehydrogenase apo form were selected as target macromolecules for molecular docking against the selected nine (Guvacine, Halostachine, Homostachydrine, Hydrocotarnine, Harmine, Piperin, Matrine, Vasicinone, and Evodiamine) natural compounds and standard drug (Diminazene). The primary objective of performing this step was to identify small molecules with satisfactory binding affinity to the selected receptors so that they could be considered for MD simulations.

Diminazene exhibited moderate binding affinity towards selected receptor macromolecules. On the contrary, our five (Harmine, Piperin, Matrine, Vasicinone, and Evodiamine) selected alkaloids revealed better binding affinity than Diminazene. Evodiamine expressed the most satisfactory docking outcome among all selected compounds, with a binding affinity of −8.7 kcal/mol for *B. microti* lactate dehydrogenase and −8.5 kcal/mol for *B. microti* lactate dehydrogenase apo form. Meanwhile, the docking outcome for Vasicinone was also noticeable as it had a binding affinity of −8.6 kcal/mol for *B. microti* lactate dehydrogenase and −7.8 kcal/mol for *B. microti* lactate dehydrogenase apo form. These two small molecules were selected for further investigation for expressing consistent results for both receptors even though Harmine, Piperin, and Matrine also conveyed promising results (**Table 2**).

**Table 2:** Binding affinity against *Babesia microti*.

Nane	*Babesia microti* lactate dehydrogenase (PDB ID 6J9D)	*Babesia microti* lactate dehydrogenase apo form (PDB ID 6K12)
	Binding affinity (kcal/mol)	Binding affinity (kcal/mol)
01 Guvacine	−5.1	−4.5
02 Halostachine	−5.3	−5.2
03 Homostachydrine	−4.9	−4.7
04 Hydrocotarnine	−5.8	−5.7
05 Harmine	−7.5	−6.8
06 Piperin	−7.6	−7.3
07 Marine	−7.4	−6.7
08 Vasicinone	−8.6	−7.8
09 Evodiamine	−8.7	−8.5
Standard Diminazene	−6.4	−6.2

### 3.3 Molecular docking pose and interaction analysis

**Figure 3:**
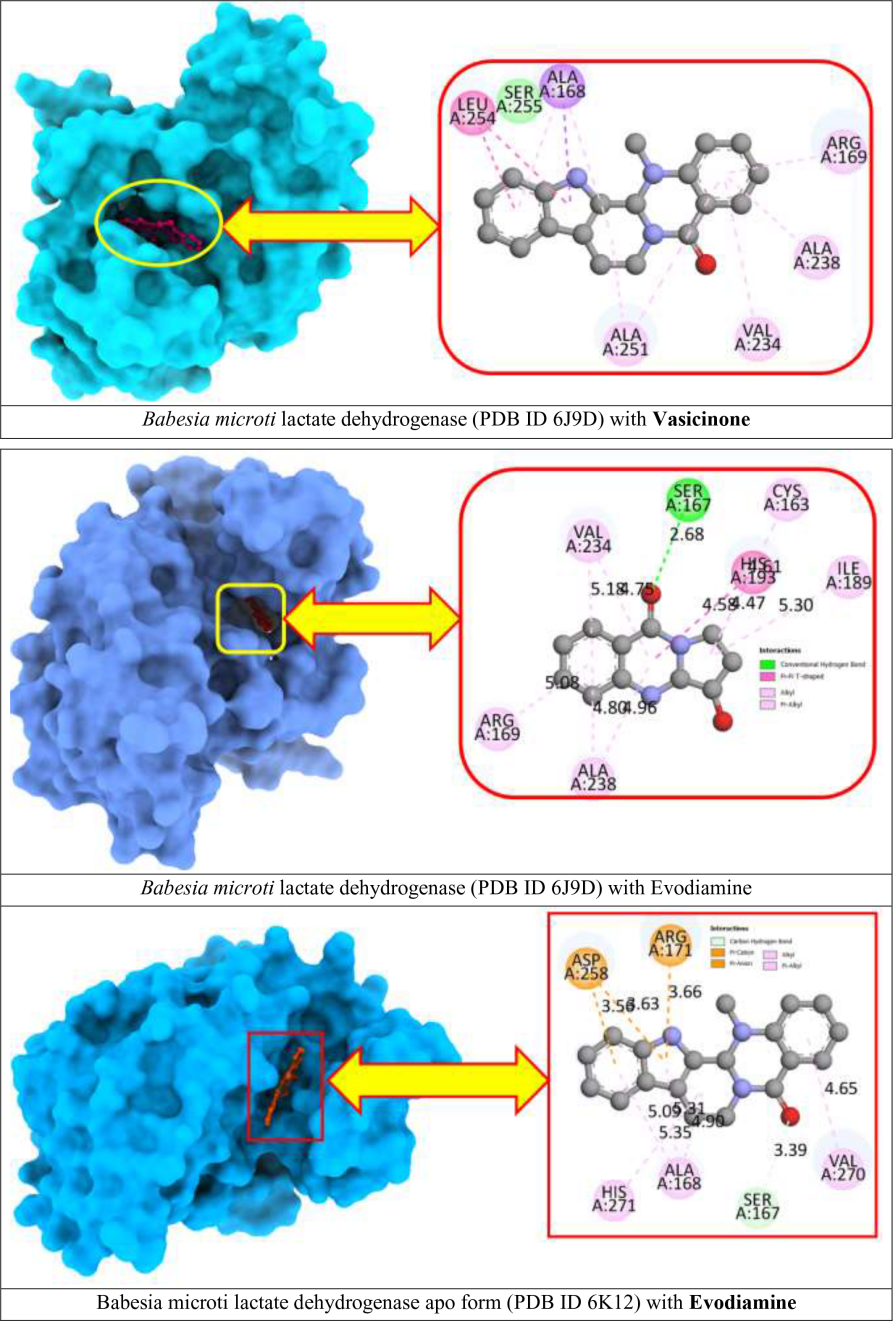
Docking interactions between the proposed compound.

To better understand the molecular interaction between selected compounds and receptor macromolecules, we decided to visualize ligand–protein docked complexes in BIOVIA Discovery Studio Visualizer. From the previous step, Vasicinone and Evodiamine expressed the strongest binding affinity for both receptor proteins since they showed the better binding energy scores. Hence, ligand–protein complexes (**Figure 3**) for these two compounds are thoroughly assessed in this step.

*B. microti* lactate dehydrogenase docked with Evodiamine showed four different types of interactions that include the following: Conventional Hydrogen Bond with SER A:167 at 2.68 Å distance; Pi-Pi T-shaped bond with HIS A:193 at 4.58 Å distance; Alkyl and Pi-Alkyl bond with CYS A:163, ARG A:169, ILE A:189, VAL A:234, and ALA A:238. On the other hand, Evodiamine docked with *B. microti* lactate dehydrogenase apo form macromolecule showed different interaction profiles with five different types of interactions. The ligand formed a Carbon–Hydrogen bond with SER A:167 at 3.39 Å distance; a Pi–Cation bond and Pi–Anion bond with ARG A:171 and ASP A:258 at reasonable distances; and Alkyl and Pi–Alkyl bond with ALA A:168, VAL A:270, and HIS A:271. Moreover, visualization for *B. microti* lactate dehydrogenase docked with Vasicinone also showed strong interactions with ALA A:168, ARG A:169, VAL A:234, ALA A:238, ALA A:251, LEU A:254, and LEU A:255.

### 3.4 Molecular dynamics simulation

To determine the stability of hit complexes, an MD study was conducted at the 100-ns simulation period. Two best-hit compounds (Vasicinone and Evodiamine) along with apoprotein and standard Diminazene were used for performing 100-ns dynamic simulation to confirm whether the ligands were sustaining at the protein’s active site. The root mean square deviation (RMSD), the radius of gyration (Rg), solvent accessible surface area (SASA), hydrogen bonds, and MMPBSA binding free energy of the hit complexes were examined.

**Figure 4:**
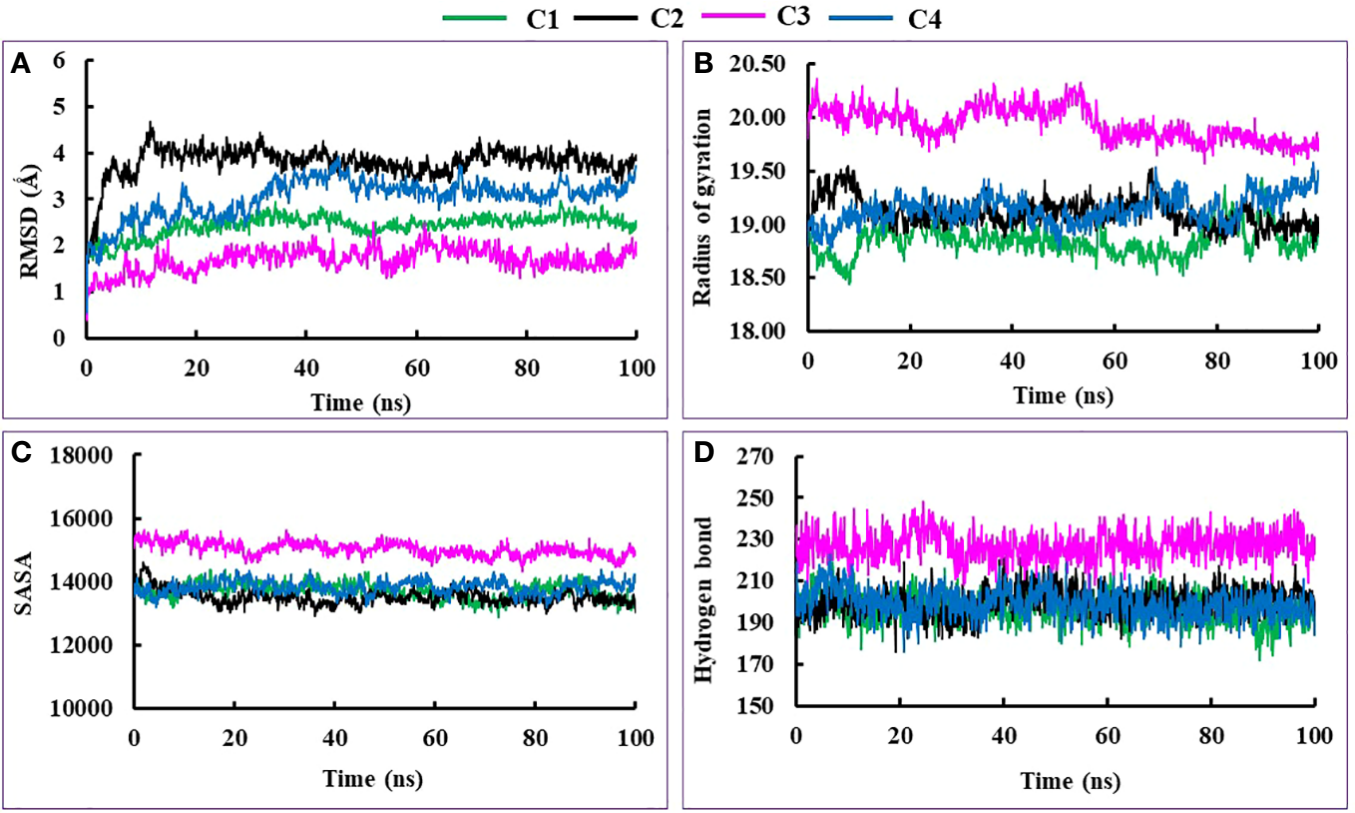
The molecular dynamics simulation of C1, C2, C3, and C4 complexes, where **(A–D)** designate RMSD, Rg, SASA, and hydrogen bond respectively.

The RMSD value is used to determine the standard measure of structural distance between coordinates. In our findings, all complexes exhibited satisfactory RMSD values with an average of 2.43, 3.80, 1.69, and 3.03 Å for C1 (Evodiamine complex), C2 (Vasicinone complex), C3 (Docked protein), and C4 (Standard Diminazene), respectively. Initially, all complexes showed increasing movement of RMSD value until 15 ns, and thereafter they maintain their stability during the simulation period with different RMSD profiles (**Figure 4A**).

During the 10 ns simulation period, the Rg profile of C2 and C4 complexes followed an increasing trend whereas C1 exhibited decreasing movement. Then, all three complexes C1, C2, and C4 showed stability and almost similar profiles until 65 ns. However, C1 and C2 met nearly the same point whereas C4 showed increasing movement at the end of the simulation period. Conversely, the C3 complex showed a higher and different Rg profile than the other three complexes although it maintains stability throughout the simulation period (**Figure 4B**).

From the very beginning to the end of the simulation period, all four complexes displayed amazing stability in terms of SASA profile; however, the C3 complex showed comparatively higher SASA value than other complexes (**Figure 4C**). Similarly, the hydrogen bond of all four complexes exhibited extraordinary stability and almost similar profile except the C3 complex during the 100-ns simulation period due to the folding and change in the confirmation of the complexes (**Figure 4**).

**Figure 5:**
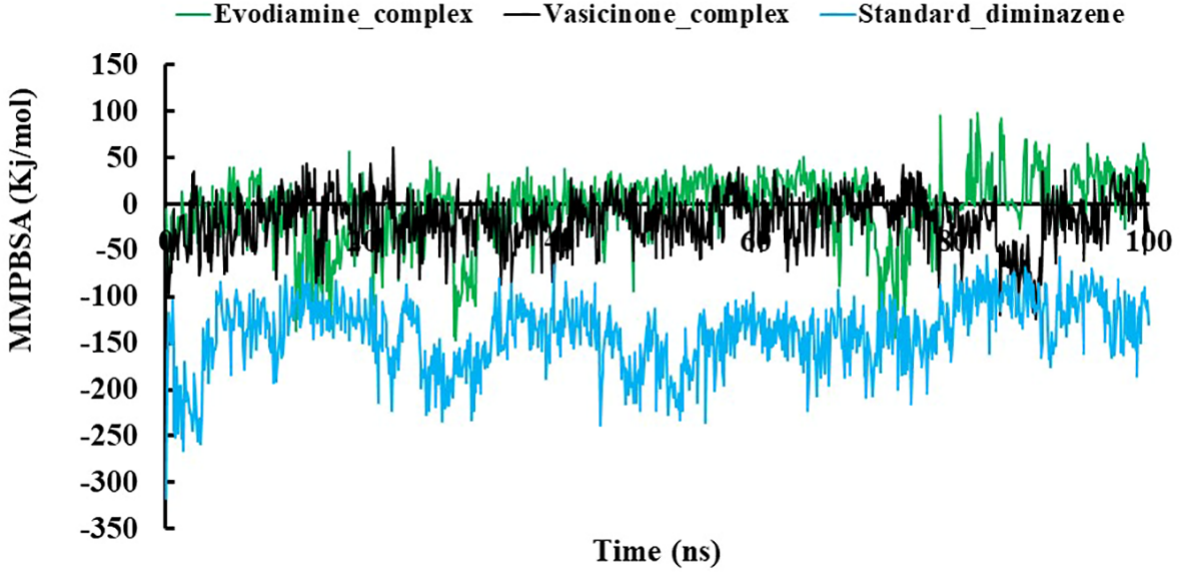
The MMPBSA binding free energy of the Evodiamine complex, Vasicinone complex, and Standard Diminazene complexes. The overall MD simulation study suggests that these two hit complexes could be used as a lead compound.

MMPBSA was used to calculate the binding free energy of interactions between protein–ligand complexes. **Figure 5** shows the MMPBSA binding free energy of three complexes at the 100-ns simulation period. The average MMPBSA binding free energies of the Evodiamine complex, Vasicinone complex, and standard Diminazene complexes were −5.294 ± 0.559, −17.576 ± 0.643, and −139.832 ± 0.824 kJ/mol, respectively (**Table 3**). The negative MMPBSA energy confirms that the ligands were sustained at the protein’s active site.

**Table 3:** The average MMPBSA binding free energy of three complexes.

Complexes	MMPBSA (kJ/mol)
Evodiamine complex	−5.294 ± 0.559
Vasicinone_complex	−17.576 ± 0.643
Standard_diminazene	−139.832 ± 0.824

### 3.5 Dynamic behavior and confirmational change of protein–ligand complex

**Figure 6:**
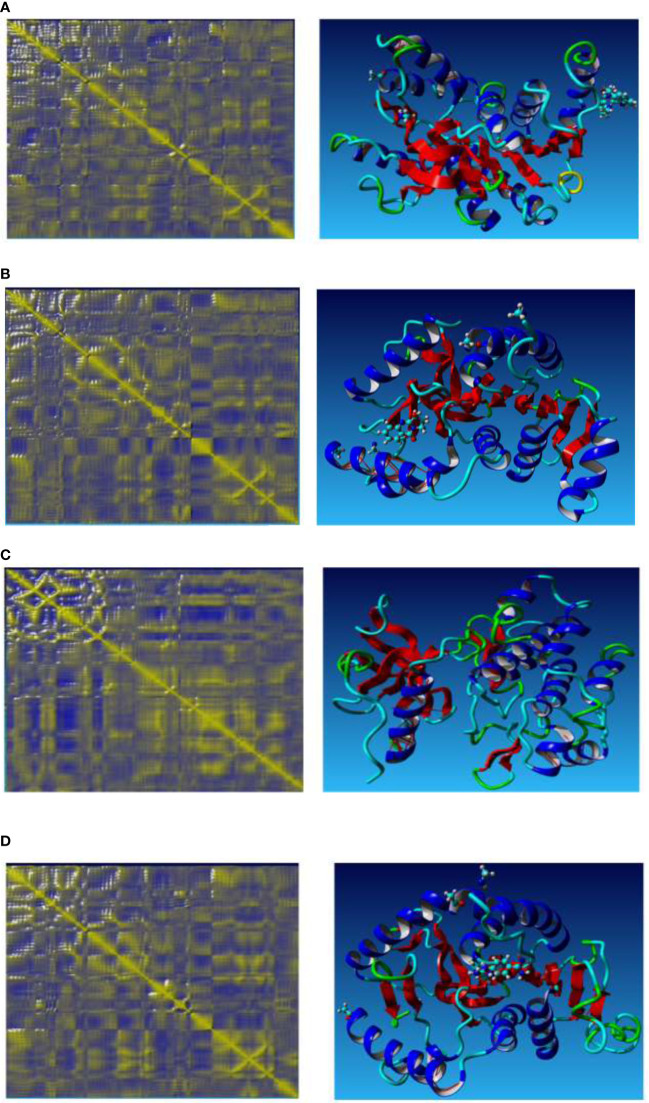
Ca-residue cross-correlation profiles for the Evodiamine complex **(A)**, Vasicinone complex **(B)**, docked protein **(C)**, and the standard Diminazene **(D)**.

Correlative motions play an essential role in recognizing and interacting with bio-macromolecular systems, which can be attained through the covariance equation of molecular perturbation generated by the MD simulation trajectory. Using the dynamic cross-correlation matrix (DCCM), the relative motions of various simulation systems were analyzed. The conformational alterations of the Evodiamine complex, Vasicinone complex, docked protein, and standard Diminazene were further analyzed using DCCM analysis. The DCCM fluctuations are illustrated in **Figure 6**, which depicts time-correlated information among the protein residues.

The greater the color intensity, the more positively or negatively correlated motions between structures are highlighted. The color yellow represents a significant positive correlation, while the color blue represents a negative correlation.

The dynamic cross-correlation map analysis was utilized to visualize the residual correlative motion in protein structures for four entities, namely, the Evodiamine complex (A), the Vasicinone complex (B), the docked protein (C), and the standard Diminazene (D). Strong correlative motions (1 to 1) are depicted on the right side of each panel using a four-dimensional caricature model. In every instance, correlations were depicted as a spectrum of colors from red to blue. The yellow color is indicative of a positive correlation between two residues, implying that both residues exhibit synchronous movement. In contrast, residues with opposite motion are designated as having anti-correlative motion and are colored blue.

The docked protein increased both correlated and anti-correlated motions in PKD1; however, it generated random motions in the majority of the crucial motifs. Positive and negative correlations were found to be stronger inbound proteins, particularly in the interloop residues and beta-sheet. The anti-correlated motion was detected in the residue range of 227 to 243 for the Vasicinone complex protein. Conversely, the Evodiamine complex protein exhibited a reduction in anti-correlated motion when compared to standard Diminazene.

### 3.6 ADMET data analysis

Predicting ADMET properties plays a critical role in ensuring the overall efficacy and safety of therapeutic molecules as more than 50% of drugs fail to reach clinical trials for not having proper ADMET attributes. pkCSM using its novel graph-based method predicted important ADMET properties for the selected therapeutic molecules (**Table 4**).

**Table 4:** Theoretical ADMET properties.

S/N	Absorption			Distribution		Metabolism		Excretion		Toxicity		
	Water solubility Log S	Caco-2 Permeability × 10^-6^	Human Intestinal Absorption (%)	VDss (human)	BBB Permeability	CYP450 1A2 Inhibitor	CYP450 2C9 Substrate	Total Clearance (ml/min/kg)	Renal OCT2 substrate	Max. tolerated dose (log mg/kg/day)	Skin Sensitization	Hepatotoxicity
01	−1.822	1.109	85.74	−0.503	−0.293	No	No	0.471	No	1.086	No	No
02	−0.717	1.567	83.23	0.806	−0.145	No	No	0.913	No	0.19	Yes	No
03	−2.374	1.442	100	−0.469	−0.469	No	No	0.551	No	0.826	Yes	No
04	−1.491	−1.491	95.08	0.655	−0.017	No	No	0.985	No	0.127	No	Yes
05	−3.476	1.594	95.14	0.364	0.53	Yes	No	0.615	No	−0.499	No	No
06	−3.841	1.893	94.35	0.268	0.274	No	No	0.24	Yes	−0.516	No	Yes
07	−2.873	1.462	94.89	0.942	0.357	No	No	0.75	No	0.21	Yes	No
08	−1.278	1.227	75.48	−0.245	−0.174	Yes	No	0.579	No	0.037	No	No
09	−4.209	1.682	93.84	−0.035	0.492	Yes	Yes	0.283	Yes	0.237	No	No
10	−3.333	−0.293	70.47	−0.157	−0.928	Yes	No	0.24	No	0.433	No	No

Drugs molecules’ absorption ability plays a critical role in their successful application. Therefore, in the absorption panel, we have paid close attention to important absorption properties such as “water solubility”, “Caco-2 permeability”, and “Human intestine absorption”. Therapeutic molecules with proper water solubility competence have high absorption in the system while the water solubility (calculated in Log S) ability can be determined from the following range: Insoluble < −10 poorly < −6, moderately < −4 soluble < −2 very < 0 < highly (Lipinski et al., 1997; Lipinski and Methods, 2000). Diminazene (compound no. 10) and compounds 03, 05, 06, and 07 were predicted as soluble in water. In addition to this, compounds 01, 02, 04, and 08 showed better water solubility than the previous group and were declared as “very soluble”. Only compound 09 was predicted as moderately soluble for having a water solubility score below −4.00 Log S. Absorption properties for orally administrated drugs are assessed by the substance’s ability to cross the Caco-2 cell line (established by Human colorectal adenocarcinoma epithelial cells) (Kumar et al., 2010). High Caco-2 permeability (value >0.9) denotes a high absorption capacity of drugs. According to the server’s predictive model, Diminazene expressed low Caco-2 permeability along with compound 04 whereas all the remaining compounds had high Caco-2 permeability. However, human intestine absorption was also predicted where Diminazene had the lowest absorption rate (70.476%). Interestingly, all our selected natural alkaloids had excellent human intestine absorption percentages (much higher than the standard drug); compound 03 had the most promising outcome (100%).

Drug distribution is the process of unmetabolized drug movement via the body’s blood and tissues. This process bears massive significance for being an indicator of the efficacy or toxicity of a drug. For the distribution panel, we have considered two properties: Volume of distribution (VDss) and Blood–Brain Barrier (BBB) permeability. A higher VDss value indicates renal failure and dehydration for the drug being distributed in the tissue rather than plasma. According to the server’s guideline, the VDss value < −0.15 indicates low, and >0.45 VDss value is considered high. Conforming to the prediction, Diminazene and compounds 01, 03, and 08 have low VDss scores while compounds 02, 04, and 07 have high VDss scores. In addition to this, compounds 05, 06, and 09 can have moderate VDss scores as their score fell between the ranges of high and low VDss scores. Our brain is protected with BBB from any non-native compounds; that is why predicting any drug’s ability to penetrate BBB could be an essential property to be considered (Daneman and Prat, 2015). Poor dispersion is indicated by a BBB permeability value of < −1, whereas a score of >0.3 denotes strong BBB permeability. Compounds 01, 02, 03, and 08 and Diminazene expressed poor BBB permeability while compounds 05, 07, and 09 were declared as high BBB permeable. The human liver contains the detoxification enzyme family, which includes cytochrome P450, which is crucial for efficient drug metabolism(Ogu and Maxa, 2000). Cytochrome P450 can be inhibited by several drugs. Therefore, it is crucial to carefully consider how well the chemicals can block this enzyme. For our experiment, the inhibition values of two cytochrome P450 isoforms—CYP450 1A2 and CYP450 2C9—were calculated. Compounds 05, 08, and 09, and Diminazene inhibited CYP450 1A2 activity while the remaining substances did not inhibit this enzyme. Besides, none of the compounds were reported to inhibit CYP450 2C9 enzyme.

Any drug-like molecules should have an acceptable excretion profile, which primarily depends on two major properties: total clearance and Renal OCT2 substrate identification. Based on the combined data of hepatic and renal clearance, total clearance produces a score and displays a distinct excretion profile for each medicine (Dowd et al., 2010). The maximum total clearance score was expressed by compound 04 and the minimum score was calculated from compound 06 and Diminazene. Meanwhile, the kidney’s ability to remove drugs, as well as additional medications from the body, depends on the renal transporter OCT2. Compounds 06 and 09 were declared as potential OCT2 substrates while the remaining compounds represented negative results.

Finally, toxicity prediction revealed that compounds 02, 03, and 07 can induce skin sensitization, and compounds 04 and 06 might provoke hepatotoxicity; therefore, precautions should be taken before applications of these selected compounds. For interpreting results for maximum tolerated dose (MTD), a value less than or equal to 0.477 indicates low MTD and a value greater than 0.477 denotes high MTD. In keeping with the given range, compounds 01 and 03 represented high MTD and the rest of the compounds had low MTD.

### 3.7 Frontier molecular orbital and molecular properties analysis

**Figure 7:**
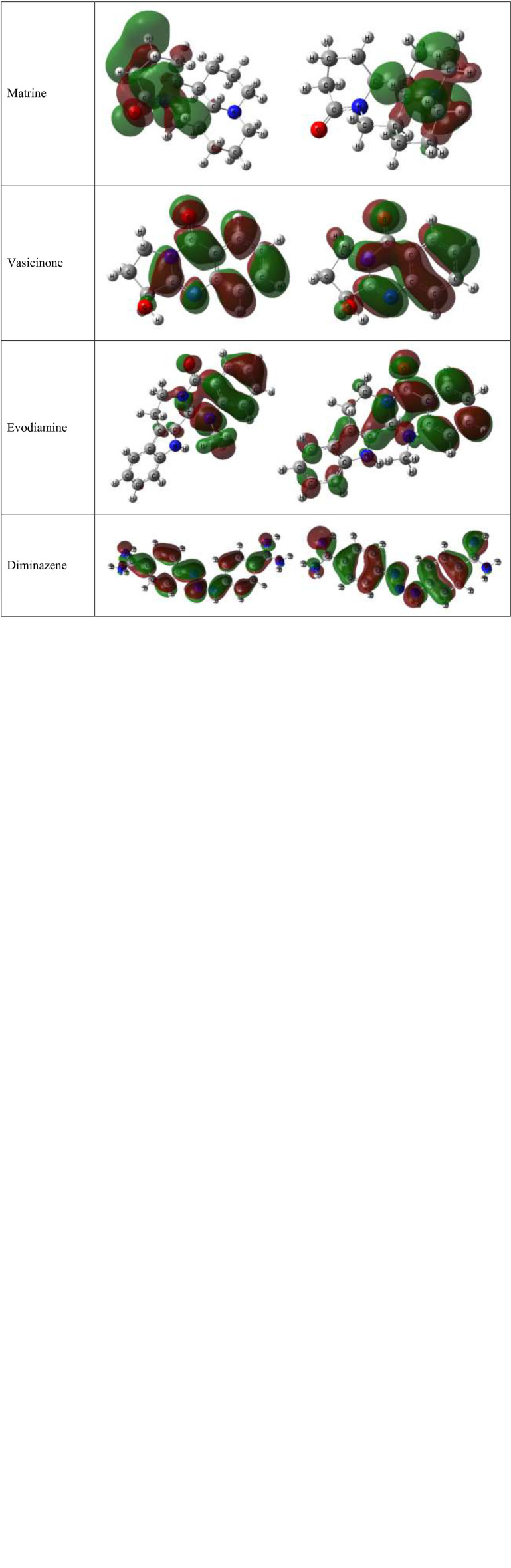
Diagram of the Frontier molecular orbital.

**Table 5** presents the HOMO–LUMO energies, hardness, softness, and energy gap of all compounds. The statistical profiles were computed utilizing the DFT function. The energy difference between the HOMO and the LUMO is a commonly employed metric for evaluating the chemical reactivity of molecules. If the energy gap between the HOMO and LUMO levels of a molecule is large, it indicates that the molecule is chemically unstable and highly unreactive. The principal cause of this phenomenon is the obstruction of the electronic transition, which is attributed to a substantial energy disparity between the ground state and the excited state. Typically, a molecule exhibiting a small HOMO–LUMO gap is indicative of a high degree of stability. The values of chemical hardness, softness, and chemical potential are contingent upon the energy of the HOMO and the LUMO. The results presented in **Table 5** indicate that the HOMO–LUMO gaps of the chemicals being studied are situated within the range of 3.1205 eV to 6.285 eV. The compounds Matrine and Evodiamine exhibited the lowest HOMO–LUMO energy gap (3.1205–3.432 eV) among the listed compounds. On the other hand, the ligand Hydrocotarnine demonstrated the highest hardness value and the widest energy gap. Typically, compounds with higher hardness values exhibit greater resistance to alterations in electron configuration at the molecular level and the obtained hardness data suggest that a prolonged period may have been necessary for the specimens to undergo disintegration after they arrived at the physiological system. Also, the ligand Piperin was found to possess compounds with a maximum softness value of 0.5435, indicating a higher rate of dissolution for this drug. The HOMO–LUMO data and Frontier molecular orbital diagram are shown in **Table 5** and **Figure 7**.

**Table 5:** Chemical reactivity and molecular properties data.

Name	I = −HOMO eV	A = −LUMO eV	Energy gap = I−A eV	Hardness	Softness
Guvacine	−5.916	−1.558	4.358	2.179	0.4589
Halostachine	−6.4096	−0.1246	6.285	3.1425	0.3182
Hydrocotarnine	−5.4979	−0.04988	5.44802	2.72401	0.3671
Harmine	−5.5762	−0.7684	4.8078	2.4039	0.4160
Piperin	−5.695	−2.0153	3.6797	1.83985	0.5435
Matrine	−5.854	−0.387	3.1205	2.7335	0.3658
Vasicinone	−6.550	−1.493	4.0215	2.5285	0.3955
Evodiamine	−5.704	−1.160	3.432	2.272	0.4401

## 4 Conclusion

An advanced computational investigation has been conducted by nine natural compounds against the pathogenic human *B. microti* parasite. In this view, the binding scores and stability of the protein–ligand complexes of selected compounds with targeted *B. microti* lactate dehydrogenase (PDB ID 6J9D) and *B. microti* lactate dehydrogenase apo form (PDB ID 6K12) have been applied, and their binding energy, bioactivity, MD simulation, and pharmacokinetic properties have been determined. In the initial stages, all the molecules were optimized by applying the DFT method. Among all the natural biomolecules, the highest value binding energy was reported for Vasicinone (−8.6 kcal/mol) and Evodiamine (−8.7 kcal/mol) against *B. microti* lactate dehydrogenase (PDB ID 6J9D) similar to these two ligands are also most potential against *B. microti* lactate dehydrogenase apo form (PDB ID 6K12). Furthermore, the anti-parasitic activity analysis exhibited that all the selected compounds had outstanding efficiency in comparison to the standard Diminazene. Molecular docking result indicated that Vasicinone and Evodiamine might play an essential role in the inhibition of *B. microti* lactate dehydrogenase and *B. microti* lactate dehydrogenase apo form and may act as potential anti-parasitic agents to fight the *B. microti* parasite. Then, the MD simulations were applied to the two best-docked compounds along with standard Diminazene for 100 ns to confirm the stability of drug–protein complexes. The stability of drug–protein complexes was predicted by analyzing RMSD, Rg, SASA, hydrogen bonds, and MMPBSA. This two-hypothetical value confirmed the atomic mobility, structural stability, and residue flexibility during the formation of drug–protein complex structures. Given the MD simulation, Vasicinone and Evodiamine have been suggested to form more stable complexes with the receptor than the standard Diminazene. We were also determined the theoretical pharmacokinetics and drug-likeness. It is confirmed that all the molecules are accepted by the Lipinski rule and have better ADMET features.

### 4.1 Limitations of the study

It is a theoretical investigation; to validate this investigation, and develop newer and safer drugs from natural sources, these phytochemical compounds must be carried out from computational (*in vitro* and *in vivo*), pre-clinical, and clinical trials, to find out their practical value.

## Data availability statement

The raw data supporting the conclusions of this article will be made available by the authors, without undue reservation.

## Author contributions

Conceptualization: SA, MEH, and SM. Methodology: SA, MEH, AK, SM, SJS, SS, and AK. writing original draft, and analysis: SA, MEH, SJS, and SM. Formal analysis: SA, AK, MEH, SS, IB, and SJ. Data curation and analysis: AK, SS, IB, and SJ. Writing and review: SA MEH, SJS and SM. Editing: HA-N, AK, and MB. Supervision: AK, SAN, and YJ, Resources, project administration, and data validation: AK, AM HA-N, and SA. All authors contributed to the article and approved the submitted version.
